# Sudden unexpected postnatal collapse and BUB1B mutation: first forensic case report

**DOI:** 10.1007/s00414-024-03231-1

**Published:** 2024-04-26

**Authors:** Massimiliano Esposito, Francesco Sessa, Chiara Nannola, Maria Serenella Pignotti, Pantaleo Greco, Monica Salerno

**Affiliations:** 1 Faculty of Medicine and Surgery, “Kore” University of Enna, Enna, 94100 Italy; 2 Department of Medical, Surgical and Advanced Technologies “G.F. Ingrassia”, University of Catania, Catania, 95121 Italy; 3 Department of Translational Medical Sciences, Università degli Studi di Napoli “Federico II”, Naples, 80125 Italy; 4 Department of Neonatology and Neonatal Intensive Care, Anna Meyer Children’s Hospital, Florence, 50139 Italy; 5 Department of Medical Sciences, Section of Obstetrics and Gynecology, University of Ferrara, Ferrara, 44121 Italy

**Keywords:** Sudden unexpected postnatal collapse, BUB1B mutation, Mosaic variegated aneuploidy syndrome, Autopsy

## Abstract

Sudden unexpected postnatal collapse (SUPC) is a sudden collapse of the clinical conditions of a full-term or near-term newborn, within the first 7 days of life, that requires resuscitation with positive ventilation and who either dies, has hypoxic-ischemic encephalopathy, or requires intensive care. The incidence of SUPC is very low, and most often presents a negative prognosis. The BUB1B gene is a mitotic checkpoint of serine/threonine kinase B that encodes a protein crucial for maintaining the correct number of chromosomes during cell division. Mutations in the BUB1B gene are linked to mosaic variegated aneuploidy syndrome 1 (MVA1), a rare autosomal recessive disorder characterized by diffuse mosaic aneuploidies involving several chromosomes and tissues. This paper discusses a case of a newborn who had a spontaneous delivery. After 2 h and 10 min, the infant showed generalized hypotonia and cyanosis, and his doctors performed orotracheal intubation, cardiac massage, pharmacological hemodynamic therapy, mechanical ventilation, antibiotic therapy, and hypothermic treatment. The newborn was discharged after 5 months with the diagnosis of hypoxic-ischemic encephalopathy. Suspecting an SUPC, a complete genetic analysis was performed demonstrating a compound heterozygous mutations in the BUB1B gene. The newborn died at 6 months of life, 1 month after discharge. A complete autopsy was performed, determining that the cause of death was due to sepsis starting from a brocopneumonic process, with outcomes of hypoxic-ischemic encephalopathy (HIE). In this scenario, it is not possible to demonstrate the causal effect of this mutation, considering that it could play a causal or concausal role in the onset of SUPC. Further research based on multicenter studies, as well as on animal models, could be very useful to clarify the pathological effect of this mutation.

**Supplementary Information:**

The online version contains supplementary material available at 10.1007/s00414-024-03231-1.

## Introduction

Sudden unexpected postnatal collapse (SUPC) is a sudden collapse of the clinical conditions of the full-term or almost full-term newborn, within the first 7 days of life, who requires resuscitation with positive ventilation and who either dies, presents hypoxic-ischemic encephalopathy, or requires therapy intensive [1]. The incidence of SUPC is very low and very often has a negative prognosis [2–4].

According to a study conducted in Karolinska University Hospital, Stockholm, Sweden, the incidence of SUPC is greatly underestimated, ranging from 2.6 cases to 133 cases/100,000. About half of the children die and of the remaining survivors half die of neurological sequelae. In more than half of cases, SUPC cannot be traced back to an etiological event [1]. The first 2 h of life are the period of greatest risk for SUPC, coinciding with breastfeeding and uninterrupted skin-to-skin contact between a mother and her newborn. SUPC is associated with prone position/skin-to-skin/co-bedding in 74% [5].

The present case report describes for the first time a case of SUPC in which an autopsy and comprehensive genetic investigation was performed and demonstrated a mutation of the BUB1B gene.

The gene family known as Budding Uninhibited By Benzimidazoles (BUB1, BUB1B, and BUB3) plays a crucial role in the spindle checkpoint during mitosis. BUB1 is essential for tasks such as chromosome congression, kinetochore localization, and establishing/maintaining efficient bipolar attachment to spindle microtubules. Acting as a paralogous gene to BUB1, BUB1B associates with unattached or incorrectly attached kinetochores, stabilizes kinetochore-microtubule attachment, and contributes to proper chromosome alignment. BUB1B is a gene involved in chromosome segregation, particularly during anaphase, through the regulation of the spindle assembly checkpoint (SAC) [6, 7]. BUB1B, therefore, is also directly implicated in chromosome alignment. In humans, a reduced expression of BUB1B is correlated with spontaneous abortions or the onset of hereditary colorectal cancer, microcephaly, intellectual disability, developmental delay and some patient-specific phenotypic abnormalities, through mosaic variegated aneuploidy (MVA) [8, 9]. The BUB1B gene mutation is extremely rare and very few studies have been published on humans. To date, there is no gender more predisposed to the BUB1 and subsequent MVA1 mutation, also because the mutation is rare and there are no studies that establish with certainty a difference in the expression of the mutation in males and females [10].

The present clinical case is the first case of SUPC related to the BUB1B mutation. Given the rarity not only of SUPC but above all of the BUB1B mutation, the aim of this case report is to inform the scientific community of the possibility of a correlation between the BUB1B mutation and the onset of a SUPC with consequences of hypoxic-ischemic encephalopathy. The genetic investigation conducted on the child was performed since it is an integral part of the clinical and forensic path in affected children who died after a SUPC [1, 11]. Furthermore, genetic investigation is crucial in cases of infant deaths, being increasingly used in forensic practices, and making an important contribution in establishing the cause of death [12–15].

## Case description

A 29-year-old woman was at her 38th week of gestation in her first pregnancy and went to the emergency department because of a ruptured membrane. The pregnancy had progressed normally, she had undergone screening procedures at the I, II, and III trimester of pregnancy as per guidelines [16], and antibiotic prophylaxis for Streptococcus agalactiae. The fetus was in a cephalic position, with normal fetal heartbeats. The cardiotocogram examination was performed and was normal. Then, the physiological birth took place with subsequent follow-ups.

The infant was born with a weight of 3860 g, length 50 cm, and head circumference 35 cm. The APGAR score was 6 in the first minute and 10 in the fifth and tenth minutes. The newborn was transferred to the ward with the mother in normal clinical conditions. After 2 h and 10 min, the infant showed generalized hypotonia, cyanosis, and his doctors performed orotracheal intubation, cardiac massage, pharmacological hemodynamic therapy, mechanical ventilation, antibiotic therapy, and hypothermic treatment. The newborn was hospitalized for 5 months and was discharged home with the diagnosis of hypoxic-ischemic encephalopathy. He died 6 months later.

### Genetic investigation

The genetic analysis was conducted at the Bambino Gesù pediatric hospital (Rome, Italy). The DNA was extracted from the peripheral blood of the proband and the parents. The analysis focused on the coding regions and exon-intron junctions (± 5 bp) of genes associated with known clinical conditions (see supplementary file). Next Generation Sequencing (NGS) was performed in trio using the Twist Custom Panel (clinical exome - Twist Bioscience) kit on the NovaSeq6000 platform (Illumina). The analytical sensitivity and specificity were > 99%. The average coverage of the sequenced regions was 257.70X. For the interpretation of the results, only regions with a minimum read depth of 30X were considered.

Sanger sequencing was then used to validate WES results. NGS data analysis was carried out using BWA (Aligner) 0.6.1-r104-tpx or DRAGEN Germline. The clinical interpretation of genomic variants was done by Geneyx Analysis (Knowledge-Driven NGS Analysis tool powered by the Gene Cards Suite). Variants were classified as pathogenic/likely pathogenic/VUS/likely benign/benign, according to the 2015 American College of Medical Genetics and Genomics (ACMG) guidelines [17]. Prediction of pathogenicity of non-synonymous variants was determined by in silico tools such as Sift, Provean, MutationTaster, and Regulation Spotter.

The sequence analysis revealed the heterozygous compound variants c.580 C > T and c.2309G > A in the BUB1B gene, which at the protein level result in the introduction of the premature stop codon p.Arg194Ter (rs28989186), and the amino acid change p.Arg770Gln (rs1422532977), respectively. The premature stop codon p.Arg194Ter (rs28989186) was found in heterozygosity in the father’s DNA, while the amino acid change p.Arg770Gln (rs1422532977) was found in heterozygous in the maternal’s DNA. The nonsense variant p.Argl94Tcr, segregating paternally, has an allelic frequency of 0.00001064 in the general population (gnomAD), is reported in scientific literature [18], and annotated in the ClinVar database (ID: 6760) and can be classified according to ACMG guidelines as pathogenic variant (class 5). The missense variant p.Arg770Gln (rs1422532977), obtained from maternal chromosome segregation, has an allelic frequency of 0.000003991 in the general population (gnomAD), is not reported in scientific literature, is annotated in the ClinVar database (ID: 1,436,353) and can be classified according to ACMG guidelines as a variant of uncertain significance (VUS) (class 3), i.e., with undefined functional and clinical effects.

### Autopsy findings

A complete autopsy was performed, and the cadaver weighed 4443 g. Neonatal growth indices were measured (Table 1). The newborn had a percutaneous endoscopic gastrostomy on the abdomen. The evisceration of the organs was performed using the Letulle technique [19]. Organs were preserved and fixed in 10% buffered formalin. On macroscopic examination the brain had a reduced consistency.

**Fig. 1:**
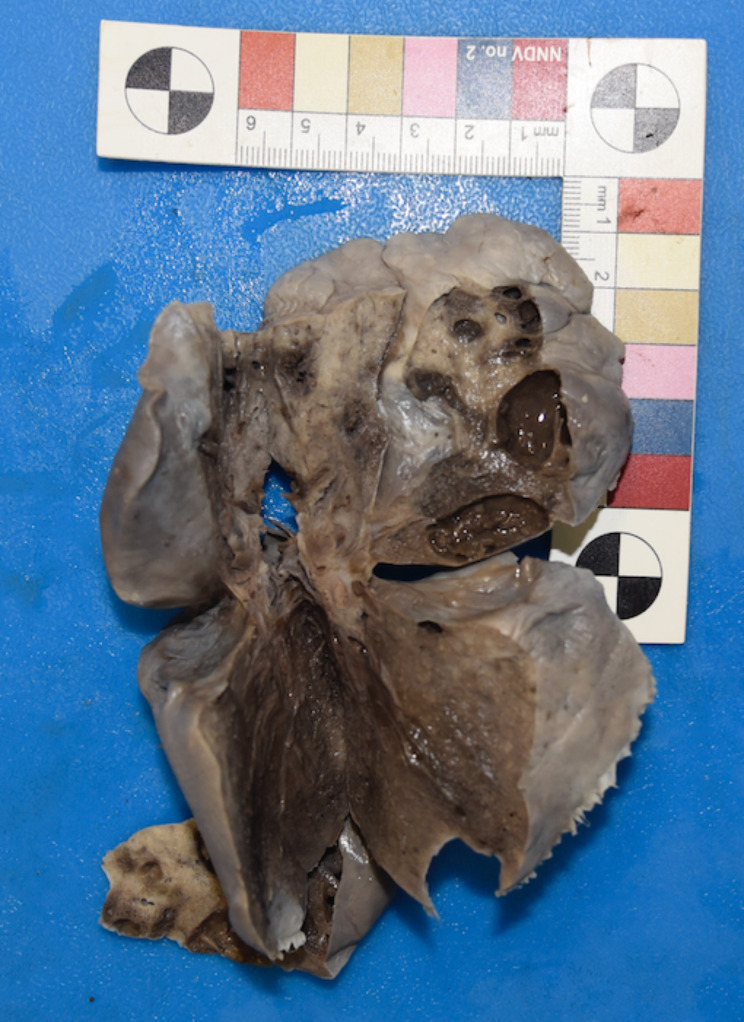
Subpleural bullae of the right lung

While the left lung measured 9.5 × 8 × 4 cm, it weighed 87 g. The right lung measured 10.5 × 7 × 3.8 cm, weighed 87 g and showed: in the middle lobe a subpleural bullae measuring 1 × 0.8 cm, in the inferior lobe subpleural bullae measuring 2 × 1.9 cm, and in the lower lobe, two subpleural bullaes 2 × 0.9 cm and 2 × 1.5 cm (Fig. 1).

**Table 1:** Neonatal growth indices

Growth measurement	Measurement (cm)
Vertex-heel length	65
Vertex-sacrum length	40
Head circumference (frontal bumps)	37
Biparietal diameter	23
Chest circumference (breast areolas)	36
Abdominal (umbilical) circumference	34
Femur length	13
Foot length	9.06
Interpupillary distance	5
Distance between medial canthi	2.05
Distance to lateral canthi	7.06
Nasolabial sulcus distance	0.8

### Histological investigation

**Fig. 2:**
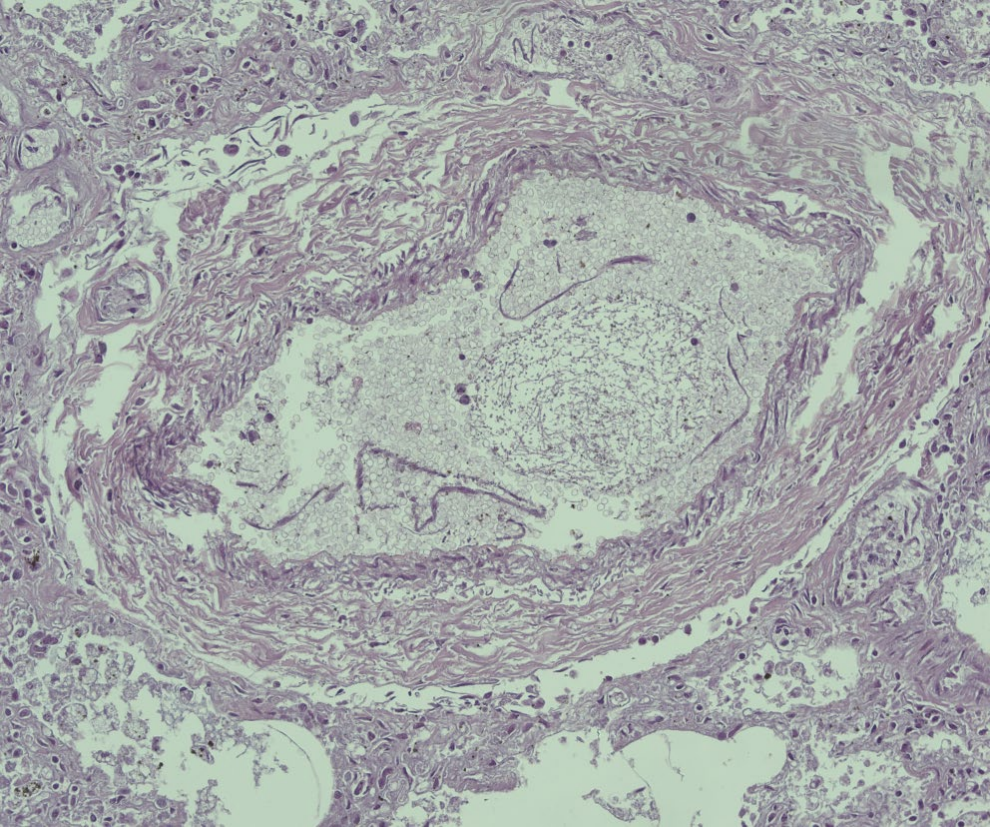
H&E 50x. Lung, septic emboli

Tissue samples were first dehydrated in graded ethanol, and then clarified in xylene and embedded in paraffin. Paraffin blocks were obtained and cut to a thickness of 4 μm, by a microtome and, finally, and sections were mounted on silane-coated slides (Dako, Glostrup, Denmark) were prepared and stored at the standard temperature room. The sections were then stained with hematoxylin and eosin (H&E). The Zeiss Axioplan (Carl Zeiss, Oberkochen, Germany) was used as an optical microscope, and then images were extracted through the Zeiss AxioCam MRc5 digital camera (Carl Zeiss, Oberkochen, Germany). The heart samples displayed foci of contraction band necrosis, and waviness. Lung sampling showed outbreaks of bronchopneumonia with septic emboli, acute emphysema, and edema (Fig. 2). Brain samples showed signs of stasis and edema. Histological samples of the liver, kidney, pancreas, intestine, spleen and adrenal glands were also performed and did not show any pathological elements.

The cause of death was due to sepsis starting from a brocopneumonic process, in a 6-month-old patient suffering from hypoxic-ischemic encephalopathy (HIE) due to SUPC.

## Discussion

SUPC is defined as the collapse of the clinical conditions of a term or near-term newborn, in good clinical condition, who has an Apgar score > 8 at the tenth minute and who, suddenly and unexpectedly, manifests apnea, acute cyanosis or paleness, requiring resuscitation support characterized by cardio-pulmonary resuscitation with, more or less, oro-tracheal intubation [20]. The British Association of Perinatal Medicine has defined SUPC as a sudden collapse of the clinical condition of the full-term or near-term newborn, who, within the first 7 days of life, assigned to routine postnatal care, requires resuscitation with positive ventilation and who either dies, or has hypoxic-ischemic encephalopathy, or requires intensive care in an NICU [21].

Most cases of SUPC occur within the first 24 h of life: 36% within the first 2 h after birth, 29% between 2 and 24 h after birth, while 24% occur between 24 and 72 h after birth and 9% between the 4th and 7th day of life. There are only approximately 2,000 cases described in the literature reporting a widely varying incidence of SUPC, with estimates ranging from 1.92 per 100,000 live births to 38 per 100,000. The prognosis is, most of the time, negative, with a mortality rate of 50% of cases and, with the same frequency, they are inexplicable [1, 22].

The timing of SUPC onset is very varied, some authors maintain that it develops in the first 24 h of life, others, however, maintain from 0 to 7 days of life, others, such as Becher et al. [23] stated that the manifestation appears within the first two hours of life in 73% of cases. A recent article reported five cases of SUPC in which all the newborns were full-term (36 to 38 weeks of gestation), born by vaginal delivery (4 cases) or by cesarean Sect. (1 case), with a collapse that occurred within 2.5 h of birth [24].

In the present clinical case, the newborn was born from a full-term pregnancy (38 + 2 weeks of gestation), with an Apgar score of 10/5 and a sudden collapse of clinical conditions 2 h after birth. Therefore, according to the criteria established by the guidelines [21], a diagnosis of SUPC was established.

One of the most common risk factors is uncontrolled skin-to-skin contact between the mother and newborn. In fact, during the first breastfeeding there is skin-to-skin contact between mother and newborn in the prone position [5]. According to some studies, however, it has not yet been clarified that this could lead to a risk factor for SUPC due to the asphyxial mechanism, in fact, some authors recommend carrying out nursing monitoring in this phase [24]. Even unmonitored skin-to-skin contact can constitute a risk factor, very little is known about the etiology of SUPC, which most of the time remains unknown, as newborns do not have problems related to prematurity and low Apgar scores at birth [20]. Becher et al. [23] state, in fact, that SUPC is rare in any center and there is no standard approach to investigations. In cases where collapse is not due to an underlying abnormality, breastfeeding and prone positioning are important associations.

In the present study, a diagnosis of SUPC was made through retrospective analysis of the medical records. Then a complete autopsy was performed showing the findings of an HIE with bronchopulmonary septic emboli. Finally, a complete genetic analysis was made that showed a mutation in the BUB1B gene.

Regarding the autopsy and histopathological-forensic findings of the outcomes of a HIE, it is consistent with other studies, since many of the newborns with SUPC die from hypoxic-ischemic encephalopathy. Indeed, Pejovic NJ et al. [7], showed that out of 26 cases of SUPC, 5 had HIE treated by hypothermia. However, to date, there are no published case reports in which a complete autopsy of a newborn with SUPC was performed, this, in fact, would be the first.

However, the finding of the BUB1B mutation has been identified for the first time. There are no studies that correlate the mutation of the BUB1B gene with SUPC, so it is not possible to establish a causal relationship between the two events, but it is important to report this to the scientific community.

Mutations in the BUB1B gene are associated with MVA1 and involve different chromosomes and tissues. MVA1 is typically related to problems during pregnancy (IUGR), neonatal (postnatal growth retardation and severe neurological damage including microcephaly) and development (developmental delay/cognitive disability, epileptic seizures and generalized hypotonia, different types of neoplasms) [25]. In mice, mutations in the BUB1B gene cause the onset of oncogenesis. A surveillance mechanism linking loss of BUB1B to the activation of the p53 pathway, promoting oncogenesis, has recently been reported [26]. Specifically, the most frequently encountered neoplasms are colorectal cancer, lung cancer, pancreatic cancer, but also thyroid follicular adenoma, leukemia, and glioblastoma [27–30]. A recent study also correlated the mutation of the BUB1B gene with the onset of pediatric neuroblastoma, bladder cancer, breast cancer, and endometrial carcinoma [31–34]. A published case report giving the clinical history of an Italian girl with a karyotype showing 12% mosaics with a new variant of the BUB1B gene (c.2679 A > T, p.Arg893Ser). The girl was two years old and presented with severe neurological disorders, microcephaly and epileptic seizures [35]. Another case report involved a child who, at 6 months of age, showed severe developmental delay, microcephaly, hypotonia, intractable seizures including infantile spasms with hypsarrhythmia, and Dandy-Walker malformation on MRI. Notably, the seizures were resistant to antiepileptic therapy and adrenocorticotropic hormone treatment. The authors concluded that knowledge of this genetic disease is important not only for a correct diagnosis but also for family genetic counseling [36]. In the present case, in fact, the child’s parents were informed during the hospital stay of the possible reappearance of the same mutation in future pregnancies, clarifying the possibility to perform a prenatal diagnosis.

In this manuscript, SUPC was followed by a 5-month hospitalization in which the infant experienced numerous healthcare-related infections from various microorganisms, including Staphylococcus epidermidis, Staphylococcus Homini, Candida Albicans, and Klebsiella Pneumoniae. Blood cultures were negative for Pneumocystis carinii. The infectious picture was also demonstrated through CT and chest x-ray. However, these infections were treated with appropriate antibiotics after susceptibility testing. After 5 months, laboratory tests were normal and the child did not have any infection but only bacterial contamination. The pulmonary findings examined showed pulmonary bulae. However, it would appear that bulae are attributable more to the long duration of hospitalization and the outcomes of nosocomial pulmonary infections, rather than to the genetic BUB1 mutation. Also Fig. 2 shows “hyphae” attributable to Candida Albicans infection.

The present case report showed the heterozygous compound variants in the BUB1B gene, which, at the protein level, result in the introduction of the premature stop codon and the amino acid change. Although the first mutation can be classified according to ACMG guidelines as a pathogenic variant, the other mutation can be classified as a VUS. In this scenario, it is not possible to demonstrate the causal effect of this mutation, considering that it could play a causal or concausal role in the onset of SUPC.

However, due to the rarity of the two pathologies (BUB1B mutation and SUPC), their relationship can be hypothesized but is not certain. Therefore, the aim of this case report is to inform the scientific community of a possible correlation between the two conditions, indicating that further studies are needed to confirm this. Moreover, in newborns who die from or following a SUPC it is necessary to perform an autopsy or a hospital autopsy complete with genetic investigation, as important elements could emerge capable of contributing to the knowledge of both the SUPC and the BUB1B mutation as well as the possible correlation between these two.

## Conclusion

SUPC is a condition characterized by sudden post-natal collapse in newborns born to full-term mothers in good clinical condition. The etiology is almost completely unknown and very few autopsies have been performed on these newborns. Unfortunately, the lack of autopsy findings in these newborns and the absence of complete genetic investigations contribute to making this pathology poorly known. The present study reports, for the first time, the autopsy and genetic findings of an infant born with SUPC who showed both HIE outcomes and a BUB1B mutation. The genetic mutation of BUB1B is an extremely rare disease associated with MVA1 that leads to the onset of serious pathologies during pregnancy, as well as during the neonatal and growth period. The only experimental studies concern mouse models, while only very few clinical cases are published on human models. This means that it is an almost completely unknown genetic mutation, especially from a clinical point of view. It is not possible to establish with certainty whether the genetic alterations detected had a causal or concausal role in the onset of SUPC as there are only reports of individual case series or studies on mouse models. However, one aspect of the case report is to describe the forensic findings of an SUPC death in a patient with a BUB1B mutation, highlighting the importance of a multidisciplinary (genetic and forensic) approach. Furthermore, the aim of the presentation is also to study the genetic mutation and its implications, also with the aim of evaluating, through follow-up, the implications in the couple’s future pregnancies.

### Electronic supplementary material

Below is the link to the electronic supplementary material.

Supplementary Material 1

## Data Availability

All data are included in the main text.
