# Supplementary figures and images for: Sudden unexpected postnatal collapse and BUB1B mutation: first forensic case report
Source: Int J Legal Med. 2024 Apr 26;138(5):2049–55. doi: 10.1007/s00414-024-03231-1 (PMC11306263; doi:10.1007/s00414-024-03231-1)

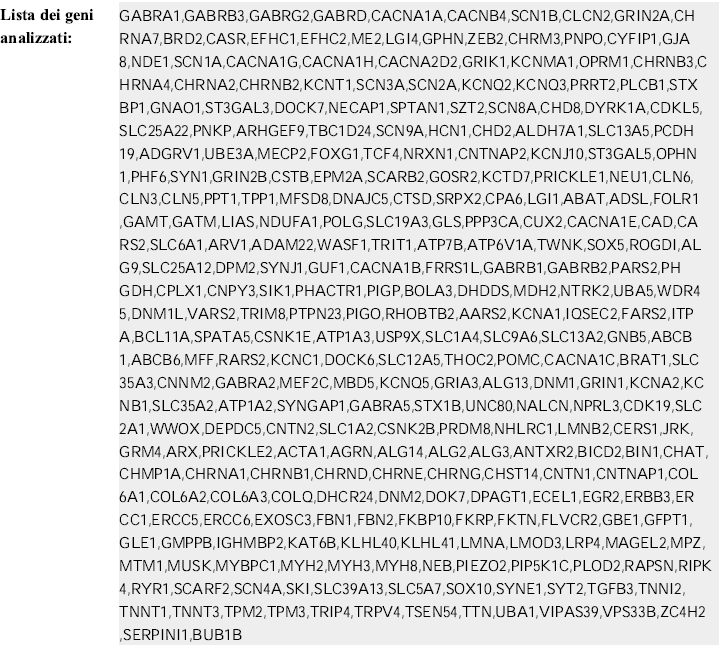


Tested genes:

Supplement: Supplementary file 1 — Supplementary Material 1 [file 414_2024_3231_MOESM1_ESM.docx]
